# New machine learning-based automatic high-throughput video tracking system for assessing water toxicity using *Daphnia Magna* locomotory responses

**DOI:** 10.1038/s41598-023-27554-y

**Published:** 2023-03-02

**Authors:** Jaehoon Kim, Hyeonseop Yuk, Byeongwook Choi, MiSuk Yang, SongBum Choi, Kyoung-Jin Lee, Sungjong Lee, Tae-Young Heo

**Affiliations:** 1 Department of Information and Statistics, Chungbuk National University, Cheongju-si, Chungbuk 28644 Republic of Korea; 2 Department of Environmental Science, Hankuk University of Foreign Studies, 81, Oe-daero, Mohyeon-myeon, Cheoin-gu, Yongin-si, Gyeonggi-do 17035 South Korea; 3 R&D Lab, Centennial Technology, Co., Ansan-si, Gyeonggi-do 15588 South Korea; 4 Engineering Division, DongMoon ENT Co., Ltd., Seoul, 08377 Korea

**Keywords:** Ecology, Environmental sciences

## Abstract

*Daphnia magna* is an important organism in ecotoxicity studies because it is sensitive to toxic substances and easy to culture in laboratory conditions. Its locomotory responses as a biomarker are highlighted in many studies. Over the last several years, multiple high-throughput video tracking systems have been developed to measure the locomotory responses of *Daphnia magna*. These high-throughput systems, used for high-speed analysis of multiple organisms, are essential for efficiently testing ecotoxicity. However, existing systems are lacking in speed and accuracy. Specifically, speed is affected in the biomarker detection stage. This study aimed to develop a faster and better high-throughput video tracking system using machine learning methods. The video tracking system consisted of a constant temperature module, natural pseudo-light, multi-flow cell, and an imaging camera for recording videos. To measure *Daphnia magna* movements, we developed a tracking algorithm for automatic background subtraction using k-means clustering, Daphnia classification using machine learning methods (random forest and support vector machine), and tracking each *Daphnia magna* location using the simple online real-time tracking algorithm. The proposed tracking system with random forest performed the best in terms of identification (ID) precision, ID recall, ID F1 measure, and ID switches, with scores of 79.64%, 80.63%, 78.73%, and 16, respectively. Moreover, it was faster than existing tracking systems such as Lolitrack and Ctrax. We conducted an experiment to observe the impact of toxicants on behavioral responses. Toxicity was measured manually in the laboratory and automatically using the high-throughput video tracking system. The median effective concentration of Potassium dichromate measured in the laboratory and using the device was 1.519 and 1.414, respectively. Both measurements conformed to the guideline provided by the Environmental Protection Agency of the United States; therefore, our method can be used for water quality monitoring. Finally, we observed *Daphnia magna* behavioral responses in different concentrations after 0, 12, 18, and 24 h and found that there was a difference in movement according to the concentration at all hours.

## Introduction

In the field of ecotoxicity, bioindicators are important in assessing whether aquatic environments are ecotoxic. Bioindicators are organisms or biological responses that reveal the presence of pollutants by exhibiting typical symptoms or measurable responses. These organisms (or communities of organisms) provide information on the changes in the environment or the quantity of environmental pollutants by changing physiologically, chemically, or behaviorally. *Daphnia magna* is a useful bioindicator in toxicology because it is sensitive to toxic substances and has a short life cycle^1^. Moreover, *Daphnia magna* is easier to culture in laboratory conditions compared with other organisms. Biomarkers, such as locomotory responses, living characteristics of *Daphnia magna* and *planktonic*, and reproductive effects of *Daphnia magna* have recently attracted considerable attention^2–4^. In particular, the locomotory responses of *Daphnia magna* have been widely used in ecotoxicology because they are sensitive to various toxicants.

The ecotoxicity of a pollutant can be measured using two indicators. The first is the median effective concentration (EC50), which is the concentration of a substance in an environmental medium expected to produce a certain effect in at least 50% of the test organisms (usually *planktonic, crustacean, and Daphnia*) in a given population under a defined set of conditions. The second is lethal concentration 50 (LC50), which is the concentration of a substance in water that results in the death of at least 50% of the tested population of the aquatic life. EC50 and LC50 are assessed using biomarkers such as the locomotory responses of *Daphnia magna*. Video-recording systems have been developed to observe the *Daphnia magna* movements. The first study involving the use of an automated video-recording system to monitor *Daphnia magna* behavior was conducted by employing a VHS video camera and tape recorder^5^. A beam was placed on a compound microscope mounted at an angle to the experiment vessel, and images were recorded at 250 frames per second at an interval of 8.7 s for 2.5 h. However, the system did not have the flexibility and simplicity of modern digital cameras. In another study, Hader and Erzinger used a 1.3-megapixel camera equipped with a macro zoom lens attached to a CS-mount lens to analyze a 50 ml cell culture flask containing 100 *Daphnia magna*^2^. Huang et al. combined a millifluidic-technology-based lab-on-a-chip device with an automated and long-term culture of *Daphnia magna*^6^. The development of a system based on video-processing technology to track *Daphnia magna* is important for aquatic toxicity assessment. Compared with manual tracking, the recorded-video-processing technology is faster and provides more accurate assessments. Tracking systems that analyze locomotory responses using video-processing technology have been widely used in recent environmental studies. For example, in several ecotoxicity studies, well-known commercial tracking systems, such as Noldus EthoVison and Lolitrack, have been employed to analyze behavioral responses^7–10^. In addition, several open-source video trackers, such as Ctrax, Tracker, and ImageJ, have been used to analyze *Daphnia magna* locomotory responses^11–13^. Such tracking systems have been employed in the environmental field as well as other fields such as agriculture, transportation, and sports^14–17^.

In general, tracking systems are divided into two parts—the part that detects bioindicators and the part that tracks each detected bioindicator. Detection is the task of detecting objects of a certain class(e.g., *Daphnia magna*, water droplets, and bioindicator waste products) within an image. Tracking is the task of creating a unique ID for each of the detected bioindicators and then tracking them in a video while maintaining their ids. In the field of ecotoxicity measurement, several tracking systems exist and are immensely effective at tracking bioindicators^7–10^. However, these systems have shortcomings in terms of accuracy and speed. The accuracy of these tracking systems is considerably affected in the bioindicator detection stage. Most detection algorithms applied in these tracking systems use a method that subtracts the background image from each frame based on the assumption that the background image is fixed. This causes new noise, such as water droplets and bioindicator waste products, to be detected along with the bioindicators. In recent studies on bioindicator-tracking systems, better performance was achieved when these systems were combined with machine learning methodologies^18–20^.

Similarly, the speed of the tracking system is affected by the tracking task. Tracking systems typically use one of two methods to track bioindicators—batch type and online type. The batch-type method measures similarity using all frames, while the online-type method measures similarity using only the before and after frames. Each type has advantages and disadvantages. The batch type is time- and resource-intensive but provides more accurate results^21–23^. By contrast, the online type consumes less time and resources but is less accurate. A simple online real-time tracking (SORT) algorithm was proposed to compensate for the shortcomings of the online-type method while maintaining lower time and resource consumption^24–26^. A deep SORT algorithm was developed to connect the similarity measure with feature maps in deep learning, and it achieved better performance than the SORT algorithm^27^.

Most studies on video tracking systems for the analysis of *Daphnia magna* focused on the development of hardware, such as building the test chamber to culture *Daphnia magna* and testing ecotoxicity^6,28^, or on the development of software that is faster and better in tracking bioindicators such as *Daphnia magna*^7–10^. However, this study aimed to develop an integrated system that focuses on building both hardware and software. Another objective was to automatically measure LC50 and EC50 using the system. To achieve the two objectives, the following steps were performed: (i) development of an automatic culture system for *Daphnia magna* and a multi-flow cell system to test the behavioral measurements; (ii) development of an automatic high-throughput tracking system using k-means clustering, machine learning, and SORT algorithms; (iii) comparison of machine learning algorithms for *Daphnia magna* detection and comparison between our tracking system and existing tracking systems, including Lolitrack and Ctrax; and (iv) application of our proposed tracking system to analyze behavioral locomotory responses of *Daphnia magna* exposed to lethal concentrations of toxicants including Potassium dichromate, Copper(II) sulfate pentahydrate, and Lead(II) sulfate, which are often found near industrial sites^6^.

## Material and methods

### Test organisms and exposures

In this study, we used test organisms and reagents according to the Acute Toxicity Test Method of *Daphnia magna Straus*(Cladocera, Crustacea); ES 04704.1b^29^. *Daphnia magna* were fostered at the National Institute of Environmental Research and were adopted. During the test, adult female *Daphnia magna* over two weeks of age, cultured over several generations, were transferred to a freshly prepared container the day before the test. *Daphnia magna* are neonates for less than 24 h after birth^29^. To maintain the sensitivity of the organism, young individuals less than 24 h old that reproduced the following day were used. Individuals of a similar size were selected for the test. *Daphnia magna* was fed YCT, which is a mixture of green algae in Chlorella sp., yeast, Cerophy II(R), and trout chow. Sufficient amounts of prey were supplied 2 h before the test to minimize the effects of prey during the test. The test medium was prepared by dissolving KCl (8 mg/L), $\text {MgSO}_4$ (120 mg/L), $\text {CaSO}_4 \cdot 2 \text {H}_2 \text {O}$ (120 mg/L), and $\text {NaHCO}_3$ (192 mg/L) in deionized water.

### Automatic high-throughput *Daphnia magna* tracking system

**Figure 1:**
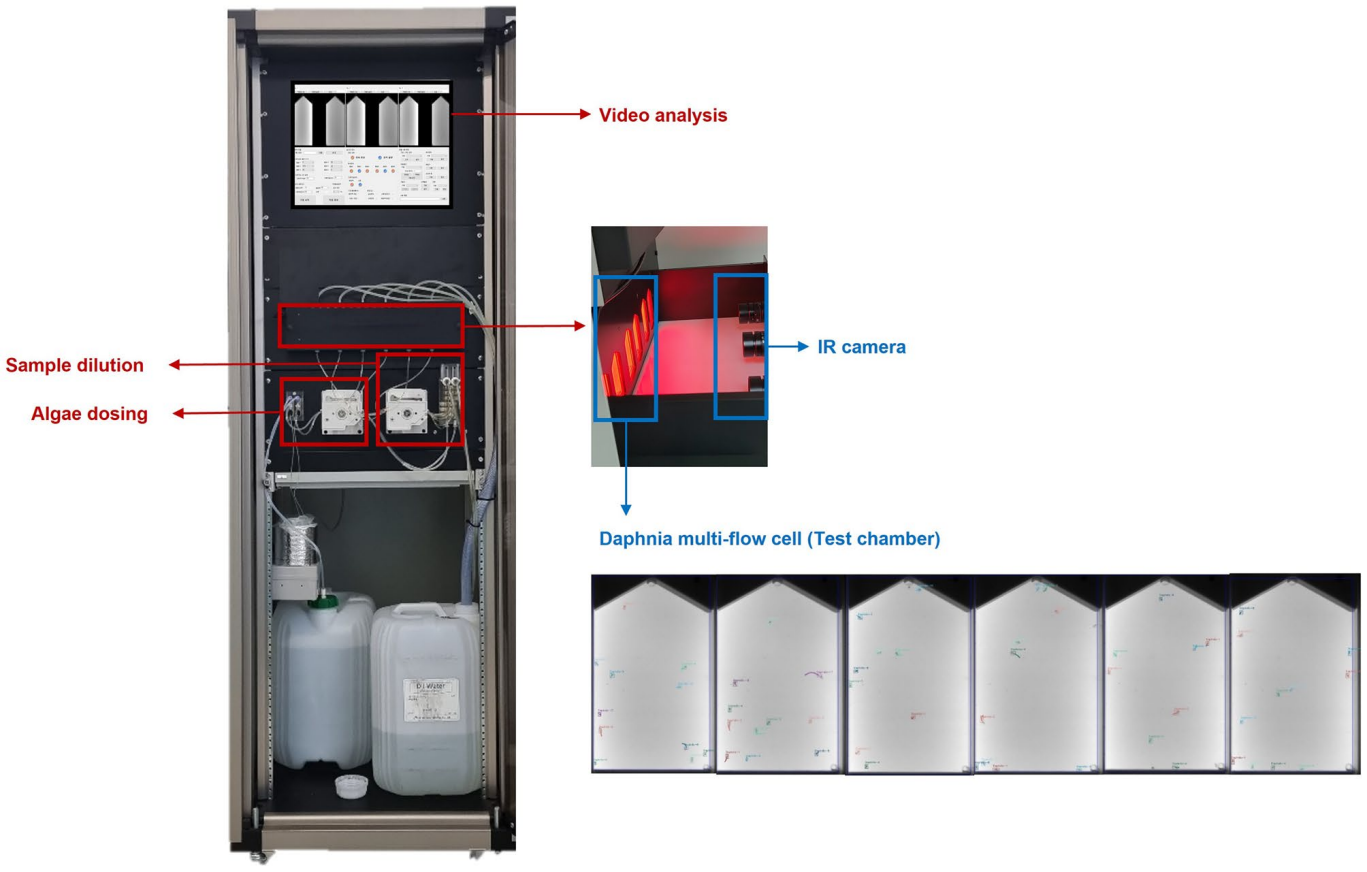
New automatic high-throughput video tracking system for behavioral analysis using *Daphnia magna* as a model organism

To build an automatic high-throughput *Daphnia magna* tracking system, we equipped the system with a video analysis algorithm as well as flow cells (Fig. 1). In the tracking system, six flow cells filled with culture medium were installed in the device. Each flow cell contained 10 *Daphnia magna*. Subsequently, to automatically measure the state of *Daphnia magna*, the six flow cells were photographed at 15 frames per second using a camera (Industrial Development Systems imaging) equipped with a CMOSIS sensor capable of infrared imaging. A red light close to the infrared spectrum was placed at the back of the flow cells for uniform illumination and to minimize stress on *Daphnia magna*. To capture the size and movement of the *Daphnia magna* as accurately as possible, the camera was set to a frame rate of 15 fps and a resolution of 2048 $\times$ 1088 (2.23 MB), using a 12 mm lens. The distance between the flow cell and the camera was set to 16 cm. To measure the number of mobile *Daphnia magna*, their lethality, and swimming inhibition automatically and simultaneously, one camera for every two cells was used to collect the status data of *Daphnia magna*. For assessing ecotoxicity, the video analysis system used images obtained from the six flow cells to track each *Daphnia magna* and estimate key statistics such as the number of mobile individuals, average distance, and radius of activity.

**Figure 2:**
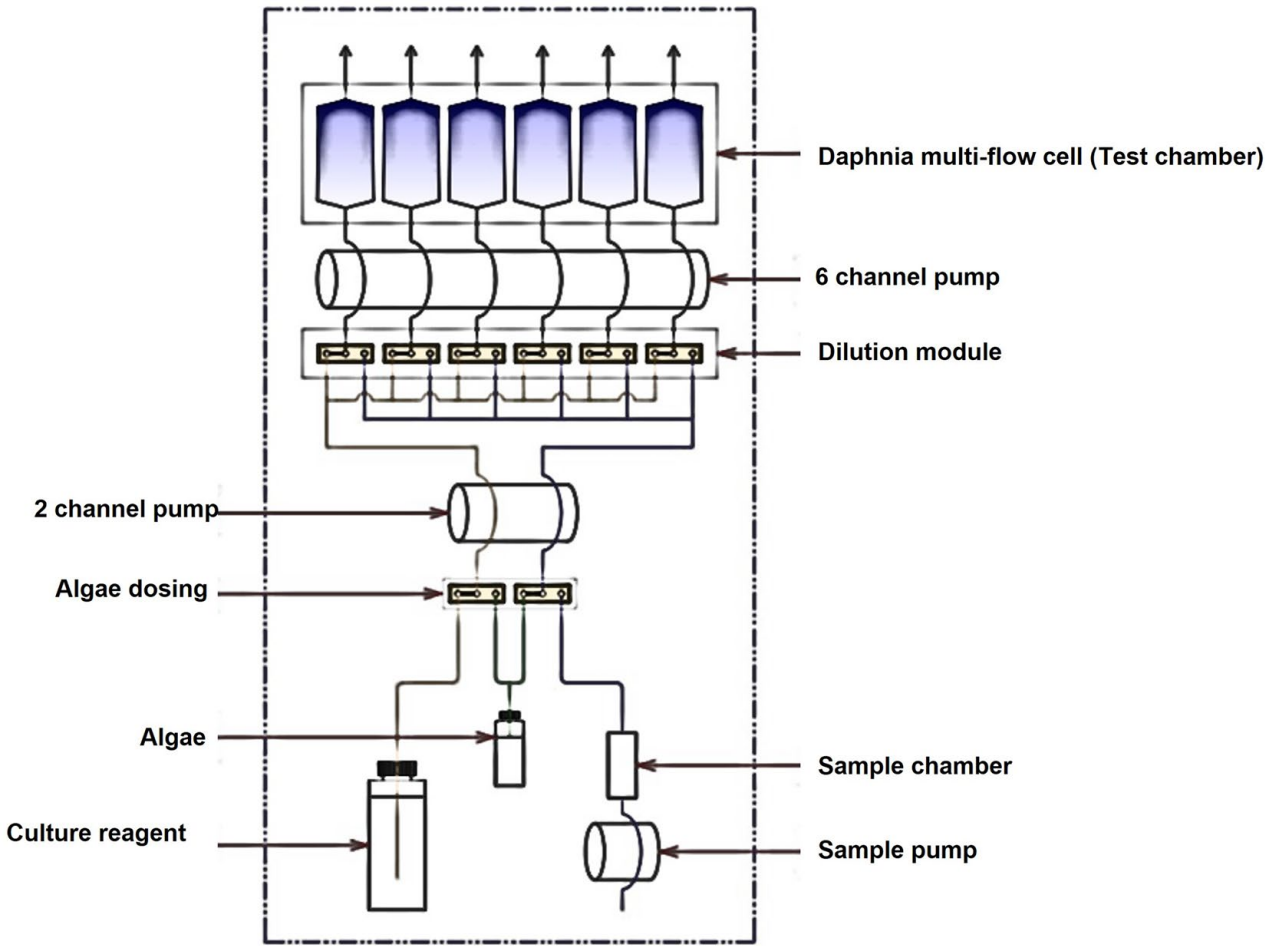
Schematic representation of the automatic high-throughput video tracking system

The automatic high-throughput video tracking system in the ecotoxicity measuring device was designed to continuously measure the ecotoxicity of *Daphnia magna* (Fig. 2). *Daphnia magna* moves faster at high temperatures and is less active at low temperatures. Thus, a constant temperature module that can be set to an appropriate *Daphnia magna* habitat temperature (20 ± 2 $^{\circ }$C) was added to create a suitable culture environment for *Daphnia magna*^29^. Natural pseudo-light ($\lambda >590$ nm, 3000 k) was installed on the upper part of the detector for proper habitat light intensity (500 Lux–1000 Lux). The size of the flow cell was set as small as possible while observing the movement of the *Daphnia magna*. An automatic feeding system was installed so that food could be injected during the replacement cycle. The six independent multi-flow cells were designed with an automatic dilution injection module; therefore, these flow cells were diluted to six different concentrations (100%, 50%, 25%, 12.5%, 6.25%, and 0%).

### Automatic tracking algorithm

**Figure 3:**
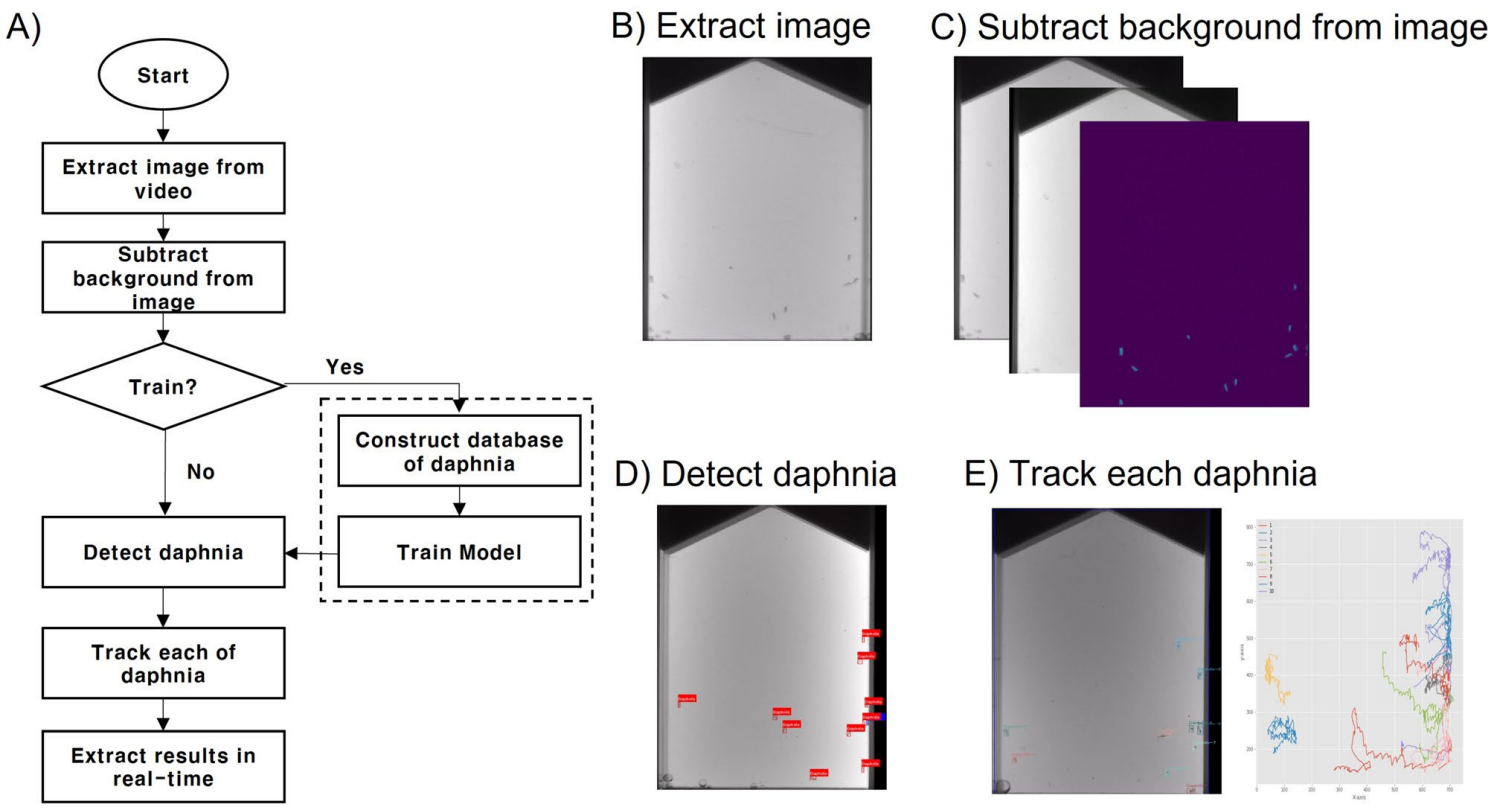
Automatic *Daphnia magna* tracking algorithm process. (**A**) Overview of automatic tracking algorithm process. (**B**) Image extraction step. (**C**) Background subtraction step. (**D**) *Daphnia magna* detection step. (**E**) *Daphnia magna* tracking step.

The CPU used for *Daphnia magna* tracking was Intel i5-9300H @ 2.40 GHz, with 8 GB of memory and Windows 10 Pro 64-bit operating system. In this experiment, the algorithms were trained using 12 *Daphnia magna* videos and tested using an additional four *Daphnia magna* videos. Subsequently, the detection and tracking methods were compared. The videos, each of which had a duration of 30 s, were captured at a rate of 15 frames per second. Generally, for long-time or real-time videos, the following factors must be considered in tracking *Daphnia magna*: automatic binarization between the object and background, effective classification of *Daphnia magna* or noise, and the speed of the algorithm. Therefore, to develop an efficient tracking algorithm, we propose the following tracking process (Fig. 3A). In this process, each frame is initially converted into an image and the background is identified from the obtained video (Fig. 3B). The background is the average of the frames over the previous 20 s, and the tracking system takes 20 s to capture the first background image. The background is subtracted from the image for object detection (Fig. 3C). The objects include *Daphnia magna* and noise such as droplets and sediment. The difference between the background and frame images is binarized, and each area of the binarized values is regarded as an object. Conventionally, the binarized values are manually generated using specific thresholds. In this study, the images are automatically binarized using k-means clustering to select the threshold value. After binarization, several machine learning methods are used to classify the objects as *Daphnia magna* or noise (Fig. 3D). For a faster tracking algorithm, we use simple machine learning methods such as random forest (RF) and support vector machine (SVM). The predicted *Daphnia magna* are tracked using SORT^24^, which is a fast and highly accurate tracking algorithm (Fig. 3E). Finally, based on the tracked results, statistics for assessing ecotoxicity, such as the number of mobile individuals, average distance, and radius of activity, are estimated to evaluate the toxicity of the aquatic environment.

#### k-means clustering for automatic background subtraction

**Figure 4:**
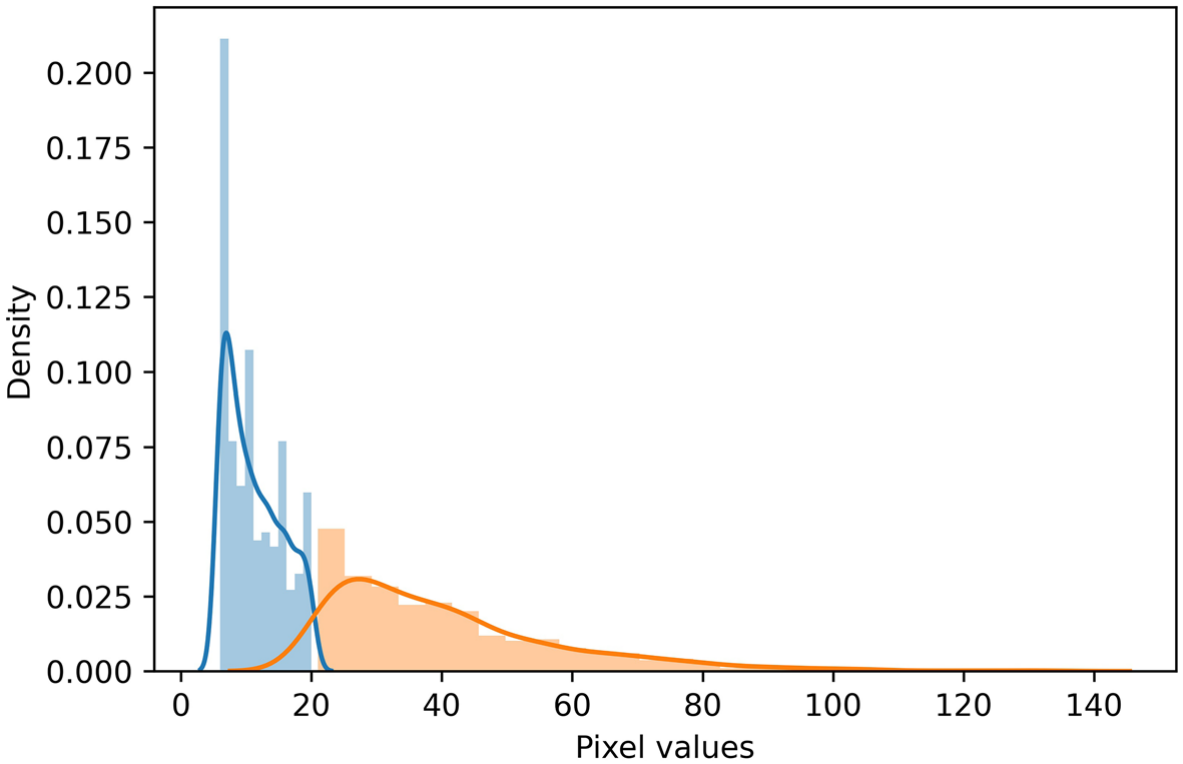
Example of automatic threshold value setting for binarization between objects and background using k-means clustering

Many tracking algorithms assume that the background is fixed. With fixed backgrounds, the difference between the frame and background can be used to identify objects. However, automatically selecting the precise threshold value for image pixel binarization becomes one of the key problems in identifying objects. The proposed method applies k-means clustering to the pixel values of the subtracted image^30^, and the center value of each calculated cluster mean is selected as the threshold value (Fig. 4). In the k-means clustering method, grouping is repeatedly performed using the distance between data points^31^. For binarization, two groups are formed. Let $\mu _1 (t)$ be the mean of pixels less than the threshold and $\mu _2(t)$ be the mean of pixels greater than the threshold. At first, $\mu _1(t), \mu _2(t)$ are randomly initialized. Subsequently, each pixel is grouped into a closer mean of each group. The above steps are repeated several times until the group experiences a few changes. Finally, the threshold is calculated as an average of the two means.

#### Classification methods

Object detection based solely on the subtraction between the background and frame images may have low accuracy. As the background in the proposed process is the average value of the frame images, noise may occur. Although this noise is removed by threshold selection in binarization, using only the threshold selection is not efficient for long or real-time videos. Therefore, additional noise must be classified and removed using machine learning models, requiring the construction of a database. In the database, the obtained objects are manually labeled as noise or *Daphnia magna* and are called ground truth. For classification, the resized 8 $\times$ 8 image of each object is stored in the database. The resized image is transformed into a feature using the Sobel edge detection algorithm^32^ and entered as inputs to the classification models. In this study, classification models such as RF^33^ SVM^34^ were used.

RF is a model that integrates several decision tree models^35^. All training data are sampled with a replacement for training each decision tree model. The decision tree model is trained to split intervals of each independent variable by minimizing the gini index (Eq. 1) or entropy index (Eq. 2). The gini index and entropy index denote the impurity within the intervals.

1 $$\begin{aligned} G= & {} 1- \sum _{i=1}^{c} p_i ^2 \end{aligned}$$

2 $$\begin{aligned} E= & {} - \sum _{i=1}^{c} p_i log_2 p_i \end{aligned}$$

where $p_i$ is a probability within *i*-th interval, and *c* is the number of intervals. For better performance, the RF selects independent variables of training data randomly. This step serves to reduce the correlation of each model. If predictions of each decision tree are uncorrelated, then the variance of an integrated prediction of models is smaller than the variance of each model. RF integrates several model predictions using the voting method. An advantage of the RF method is that it avoids overfitting because the model uses the average of many predictions.

SVM is a model designed to search for a hyperplane to maximize the distance, or margin, between support vectors. The hyperplane refers to the plane that divides two different groups, and the support vector represents the closest vector to the hyperplane. Let $D=({\textbf{x}}_i, y_i), i=1, \ldots , n, {\textbf{x}}_i \in {\mathbb {R}}^p, y_n \in \{ -1,1 \}$ be training data. Suppose that the training data are completely separated linearly by a hyperplane; then, the hyperplane is expressed as Eq. 3.

3 $$\begin{aligned} {\textbf{w}}^T {\textbf{x}} + b = 0, \end{aligned}$$

where ${\textbf{w}}$ is a weight vector of the hyperplane, and *b* is a bias. The weight vector is updated by minimizing Eq. 4.

4 $$\begin{aligned} L = {1 \over 2} {\textbf{w}}^T {\textbf{w}} \text { subject to } y_i ({\textbf{w}}^T {\textbf{x}} + b) \ge 1 \end{aligned}$$

We can transform Eqs. 4 to  5 by using the Lagrange multiplier method.

5 $$\begin{aligned} L^* = {1 \over 2} {\textbf{w}}^T {\textbf{w}} - \sum _{i=1}^n a_i \{ y_i ({\textbf{w}}^T x_i + {-}) - 1 \}, \end{aligned}$$

where $a_i$ is the Lagrange multiplier. We can efficiently solve Eq. 5 using a dual form. Furthermore, Eq. 5 can be solved in a case where it is not completely separated using a slack variable and a kernel trick can be used to estimate the nonlinear hyperplane.

#### SORT tracker

SORT, one of the frameworks for solving the multiple object tracking (MOT) problem, aims to achieve efficient real-time tracking^24^. The SORT method framework is created by combining the estimation step and the association step. The estimation step forecasts the next position of each predicted *Daphnia magna*. The association step matches the forecasting position and next true position of each predicted *Daphnia magna*. In the estimation step, the SORT framework uses the Kalman filter to forecast the position of the predicted *Daphnia magna* in the next frame. The position of each predicted *Daphnia magna* is expressed as Eq. 6.

6 $$\begin{aligned} {\textbf{x}} = [u,v,s,r,{\dot{u}}, {\dot{v}}, {\dot{s}}]^T \end{aligned}$$

where *u* and *v* are the center positions of each predicted *Daphnia magna*, *s* is the scale size of the bounding box, and *r* is the aspect ratio of the bounding box. ${\dot{u}}$, ${\dot{v}}$, and ${\dot{s}}$ are the amounts of change in each variable. In the association step, to associate the forecasting position and true position, the framework adopts the intersection-over-union (IOU)^36^ as the association metric. The Hungarian algorithm is loaded into the SORT framework to perform fast and efficient *Daphnia magna* association prediction. In this study, a mixed metric of IOU^36^ and Euclidean distance^37^ was used instead of only the IOU that is used in SORT (Eq. 7) for more efficient association.

7 $$\begin{aligned} C_{ij} = (1-\lambda ) {max_d - d_{ij} \over max_d} + \lambda \cdot IOU_{ij} \end{aligned}$$

where $d_{ij}$ is the Euclidean distance between the i-th predicted *Daphnia magna* in the before frame and the j-th predicted *Daphnia magna* in the next frame, and $\lambda$ is the weight of $IOU_{ij}$. $IOU_{ij}$ is the IOU between the i-th predicted *Daphnia magna* in the before-frame and the j-th predicted *Daphnia magna* in the next frame.

#### Metrics

The binary confusion matrix consists of true positive (TP), true negative (TN), false positive (FP), and false negative (FN)^38^. TP is the number of cases where the predicted *Daphnia magna* matches the actual *Daphnia magna*, TN is the number of cases where the objects predicted as noise are actual noise, FP is the number of cases where the predicted *Daphnia magna* differs from the actual *Daphnia magna*, and FN is the number of cases where the objects predicted as noise are not actual noise. In this study, accuracy, recall, precision, and F1 scores (Eq. 8) were used as the metrics for comparing the machine learning methods.

8 $$\begin{aligned} \begin{aligned} Accuracy&= {TP + FP \over TP + TN + FP + FN} \\ Recall&= {TP \over TP + TN} \\ Precision&= {TP \over TP + FP} \\ F1 \ score&= 2 \times {Precision \times Recall \over Precision + Recall} \end{aligned} \end{aligned}$$

Standard MOT metrics to evaluate tracking performance include multi-object tracking accuracy (MOTA) and multi-object tracking precision (MOTP). An important task of MOT is to identify and track the same object across two frames. Identification (ID) precision (IDP), ID recall (IDR), ID F1 measure (IDF1), and ID switches (IDs) may be used as measures for evaluating the identification and tracking of the same objects^39,40^.

### Data analysis

The toxicity test using *Daphnia magna* was performed following the Korean official Acute Toxicity Test Method^29^. The test medium was prepared by dissolving KCl (8 mg/L), $\text {MgSO}_4$ (120 mg/L), $\text {CaSO}_4 \cdot 2 \text {H}_2 \text {O}$ (120 mg/L), and $\text {NaHCO}_3$ (192 mg/L) in deionized water. Considering that *Daphnia magna* are neonates for less than 24 h after birth^29^, five neonates were exposed to 50 mL of different concentrations of heavy metals such as Potassium dichromate, Copper(II) sulfate pentahydrate, and Lead(II) sulfate (6.25, 12.5, 25, 50, and 100%) and 50 mL of culture media. Potassium dichromate is a common inorganic reagent used as an oxidizing agent in chemical industries. Copper(II) sulfate pentahydrate is a trace material widely used in industrial processes and agriculture. A significant amount of copper is emitted in semiconductor manufacturing processes, which adversely impacts the aquatic ecosystem. When present as an ion in water, copper can be acutely toxic to aquatic organisms such as *Daphnia magna*. Lead(II) sulfate is another nonessential and nonbiodegradable heavy metal. It is highly toxic to numerous organisms even at low concentrations and can accumulate in aquatic ecosystems^41^. Twenty *Daphnia magna* (four replicates of five each) were exposed to each test solution for 24 h. The term “immobility” means that the *Daphnia magna* remains stationary after exposure to chemicals such as Potassium dichromate, Copper(II) sulfate pentahydrate, and Lead(II) sulfate. In this study, immobility was used as an endpoint identifier, and the number of mobile *Daphnia magna* were counted to evaluate the EC50 values for the samples using the ToxCalc 5.0 program (Tidepoll Software, USA).

The locomotory responses of *Daphnia magna* were tested after 0, 12, 18, and 24 h of exposure at different concentrations. Potassium dichromate ($\text {K}_2\text {Cr}_2\text {O}_7$) at 2 mg/L was connected to the *Daphnia magna* tracking system, and standard toxic substances were automatically diluted to 100%, 50%, 25%, 12.5%, and 6.25%. The automatic high-throughput *Daphnia magna* tracking system automatically measured the tracking results of a 1-minute-long video at hourly intervals. The average moving distance for 20 s of each *Daphnia magna* in each chamber was analyzed using a repeated measures ANOVA (RMANOVA). RMANOVA was used for the analysis of data obtained by repeatedly measuring the same *Daphnia magna*^42^. It analyzes the concentration effect excluding the time effect at each hour. The time effect means the change in average distance per 20 s. RMANOVA was implemented using the agricolae package of the R 4.0.4 program^43^. To remove the noise affecting RMANOVA, the *Daphnia magna* that remained stationary for 20 s or more were removed from the observations. In this study, we used the significance level at 5%.

## Results

### Classification and tracking results

**Figure 5:**
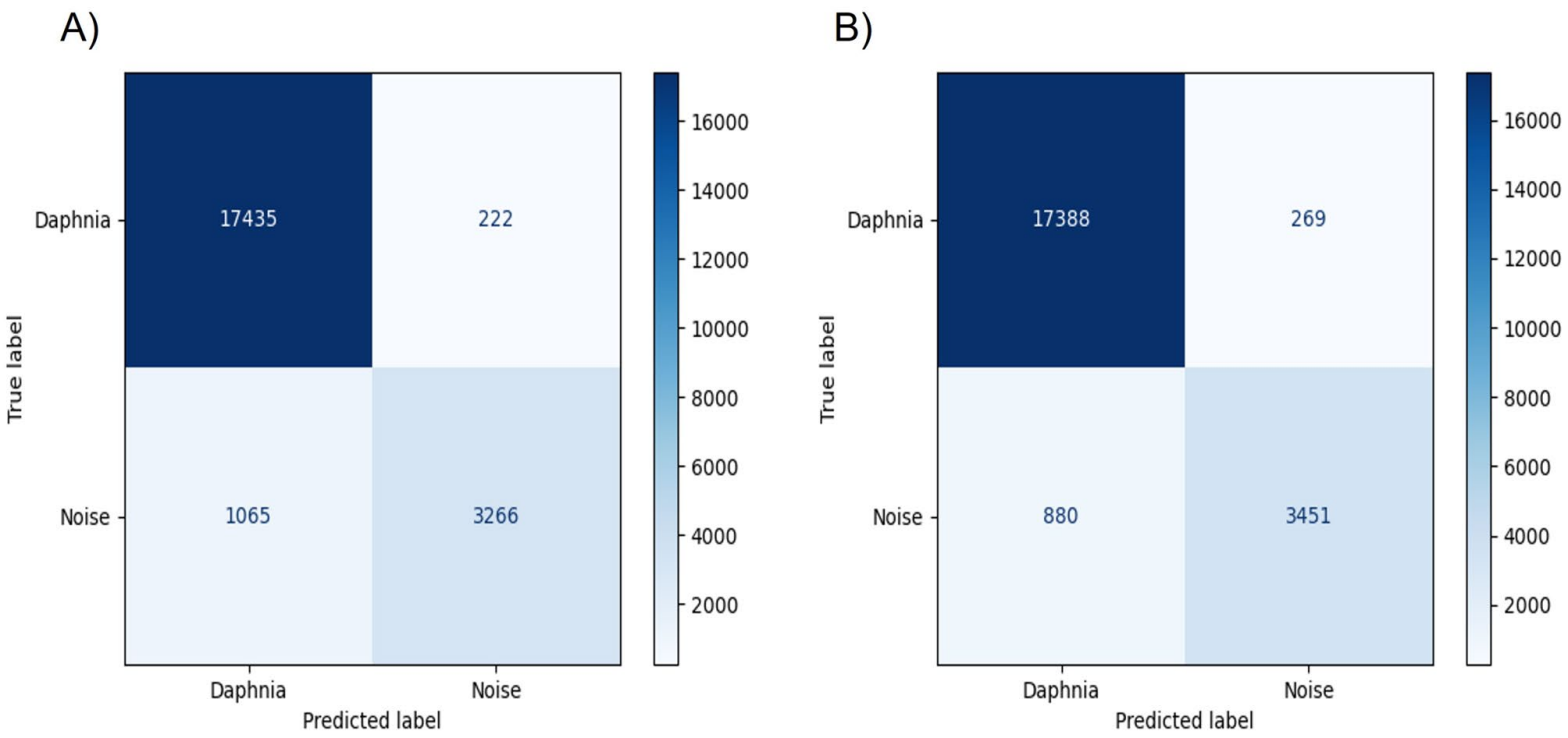
Confusion matrix of machine learning methods. (**A**) Random Forest. (**B**) Support Vector Machine

Using machine learning algorithms, the detected object for each frame was classified as noise or *Daphnia magna*. The RF method yielded accuracy, recall, precision, and F1 score of 0.841, 0.987, 0.942, and 0.964, respectively (Fig. 5A). Similarly, the accuracy, recall, precision, and F1 score of the SVM method were 0.831, 0.985, 0.952, and 0.968, respectively (Fig. 5B)

The video tracking system performed object detection using a combination of the subtraction method and the machine learning methods. An MOT system using the SORT algorithm was implemented to compare the tracking performance of Lolitrack, Ctrax, and our tracking method. Lolitrack, a commercial program, performed the best with a MOTA of 80.00% and MOTP of 75.85%. However, our proposed method using RF achieved the best performance in terms of IDF1 (79.65% ), IDP (80.63%), IDR (78.73%), and IDs (16) (Table 1).

**Table 1:** Comparison of tracking methods

Method	IDF1 (%)	IDP (%)	IDR (%)	IDs	MOTA (%)	MOTP (%)
ours_RF	**79**.**64**	**80**.**63**	**78**.**73**	**16**	76.33	75.50
ours_SVM	78.15	79.33	77.05	17	75.35	75.43
Lolitrack	66.65	66.63	66.65	25	**80**.**00**	**75**.**85**
ctrax	31.25	30.43	32.23	305	62.38	74.68

Significant values are in bold.

**Figure 6:**
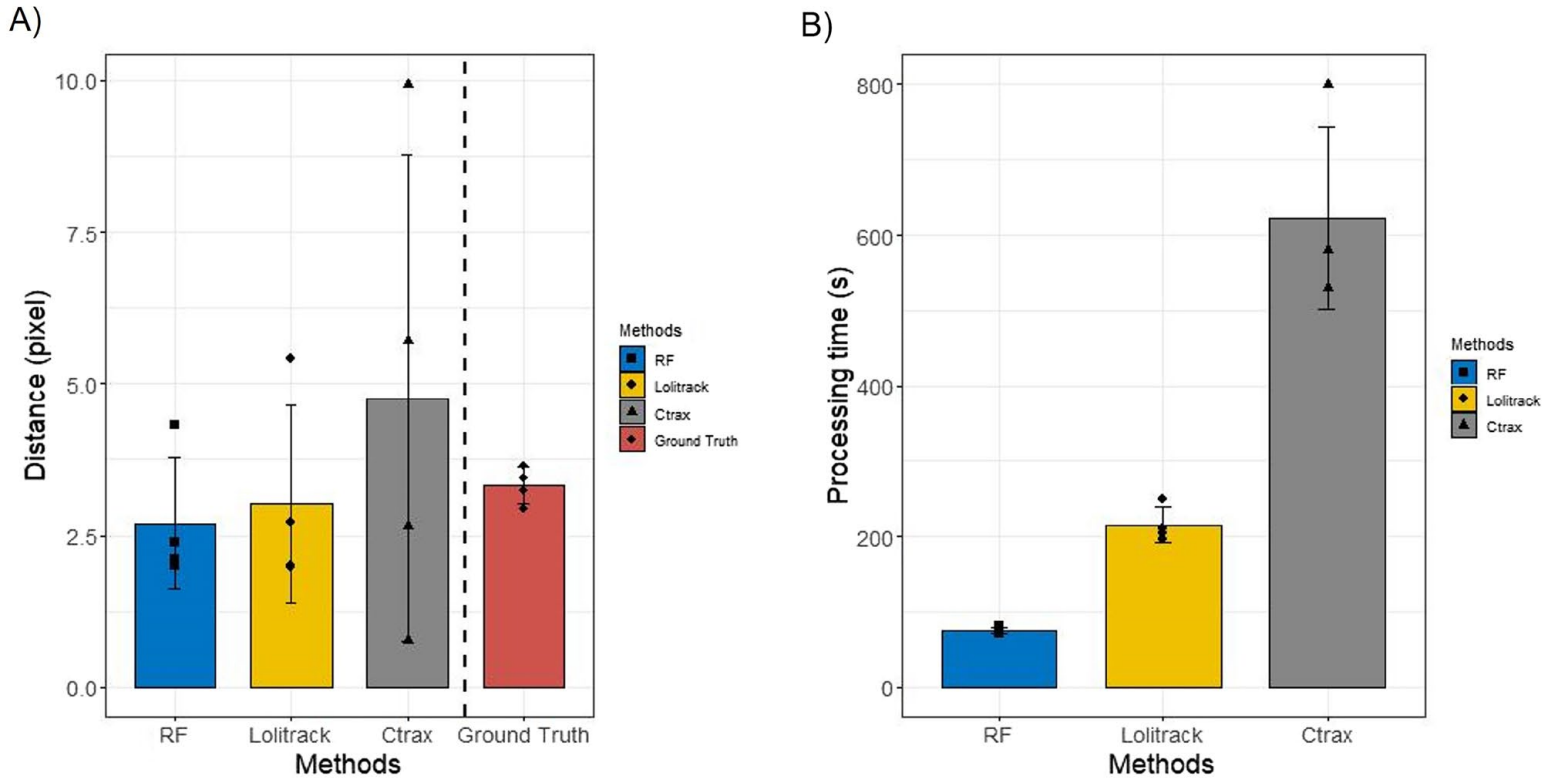
Comparison of different tracking methods. (**A**) Comparison of average distances of different models. (**B**) Comparison of processing times of different models

The average distance was calculated based on the Euclidean distance between the center position of each *Daphnia magna* in the current frame and that in the next frame. The tracking methods were compared based on this average distance (Fig. 6A). The ground truth distance values were 3.26, 3.45, 2.94, and 3.65. The mean and standard error (SE) of the ground truth were approximately 3.3 and 0.151, respectively. The average distances of the proposed method were 4.32, 2.0, 2.1, and 2.39, and the mean and SE of the distance were 2.70 and 0.545, respectively. The results of Lolitrack were 2.71, 1.97, 2.0, and 5.42. The corresponding Ctrax values were 6.77, 2.64, 9.93, and 5.71. The mean of the Lolitrack values was 3.02, which was the closest to the mean of the ground truth values. The proposed method had the least SE.

Processing time is the amount of time required to analyze each 30 s video and was used to compare the tracking methods (Fig. 6B). The processing times for the proposed method were 81, 72, 74, and 75 s, and the average time was 75.5 s. The processing times for Lolitrack were 250, 210, 197, and 205 s, and the average time was 215.5 s, and those of Ctrax were 530, 581, 800, and 580 s. Therefore, the proposed method was the fastest.

### Impact of toxicants on behavioral responses

#### EC50

The toxicity test using *Daphnia magna* was performed according to the Acute Toxicity Test Method of ES 04704.1b^29^. The test medium was prepared by dissolving KCl (8 mg/L), $\text {MgSO}_4$ (120mg/L), $\text {CaSO}_4 \cdot 2 \text {H}_2 \text {O}$ (120 mg/L), and $\text {NaHCO}_3$ (192 mg/L) in deionized water. The following heavy metals were used: Potassium dichromate ($\ge$99%, Sigma-Aldrich, Germany), Copper(II) sulfate pentahydrate ($\ge$98%, Sigma-Aldrich, Germany), Lead(II) sulfate ($\ge$ 98%, Sigma-Aldrich). Milli-Q water (Super Q-treated, Millipore, MA, USA) was used as a solvent, culture medium, and test solution in all experimental procedures. The 100% chemical solution was prepared to have 5, 1, and 1 mg of Potassium dichromate, Copper(II) sulfate pentahydrate, and Lead(II) sulfate, respectively, in 1 L of *Daphnia magna* culture medium.

**Figure 7:**
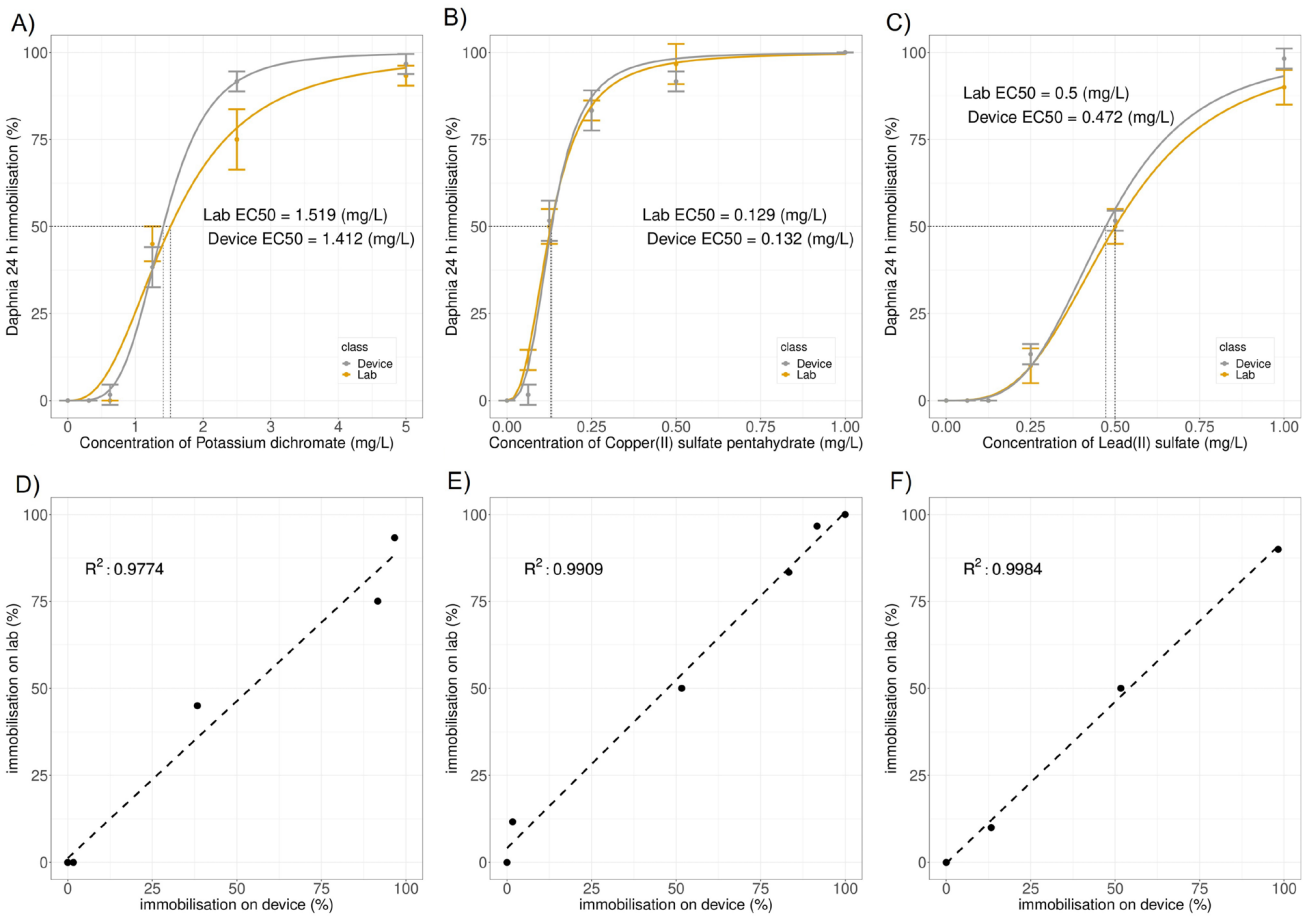
Comparison of *Daphnia magna* 24 h acute toxicity test in laboratory (Lab) and *Daphnia magna* tracking system analysis (Device) on heavy metals. Each flow cell contains 10 *Daphnia magna* and the total number is 60. (**A**) Dose-response curves of Lab and Device experiments on Potassium dichromate. (**B**) Dose-response curves of Lab and Device experiments on copper. (**C**) Dose-response curves of Lab and Device experiments on lead. (**D**) Pearson linear correlation analysis between Lab and Device on Potassium dichromate. (**E**) Pearson linear correlation analysis between Lab and Device on copper. (**F**) Pearson linear correlation analysis between Lab and Device on lead

The EC50 results of the 24 h *Daphnia magna* acute toxicity test of Potassium dichromate performed in the lab using and using the device were 1.519 mg/L and 1.414 mg/L, respectively (Fig. 7A). Potassium dichromate is classified as a standard toxic substance by the Environmental Protection Agency of the United States, and the results of this experiment satisfy the toxicity range suggested in the guidelines of the Environmental Protection Agency of the United States (EC50 0.9–2.1 mg/L). The correlation between the toxicity evaluations of the laboratory and those of the device, $\text {R}^2$, was 0.977 (Fig. 7D), showing that the relationship between the toxicity evaluations of the laboratory and the device was close.

The EC50 results of the 24 h *Daphnia magna* acute toxicity test of Copper(II) sulfate pentahydrate performed in the lab and using the device were 0.129 mg/L and 0.132 mg/L, respectively (Fig. 7B). The $\text {R}^2$ value of the toxicity evaluation of the laboratory and that of the device was 0.991 (Fig. 7E), indicating a close relationship.

The EC50 results of the 24 h *Daphnia magna* acute toxicity test of Lead(II) sulfate in the lab and using the device were 0.5 mg/L and 0.472 mg/L, respectively (Fig. 7C). The correlation between the toxicity evaluation of the lab and that of the device $\text {R}^2$ was 0.998 (Fig. 7F), indicating a very close relationship.

#### *Daphnia magna* locomotory response analysis using Repeated Measures ANOVA

We tested the locomotory responses of *Daphnia magna* after 0, 12, 18, and 24 h of exposure at different concentrations. Potassium dichromate at 2 mg/L was connected to the automatic high-throughput *Daphnia magna* tracking system, and standard toxic substances were automatically diluted to 100%, 50%, 25%, 12.5%, and 6.25%. The automatic high-throughput *Daphnia magna* tracking system automatically measured the tracking results of the 1 min long video hourly. The average moving distance for 20 s of each *Daphnia magna* in each chamber was analyzed using RMANOVA with significance level of 5%.

**Figure 8:**
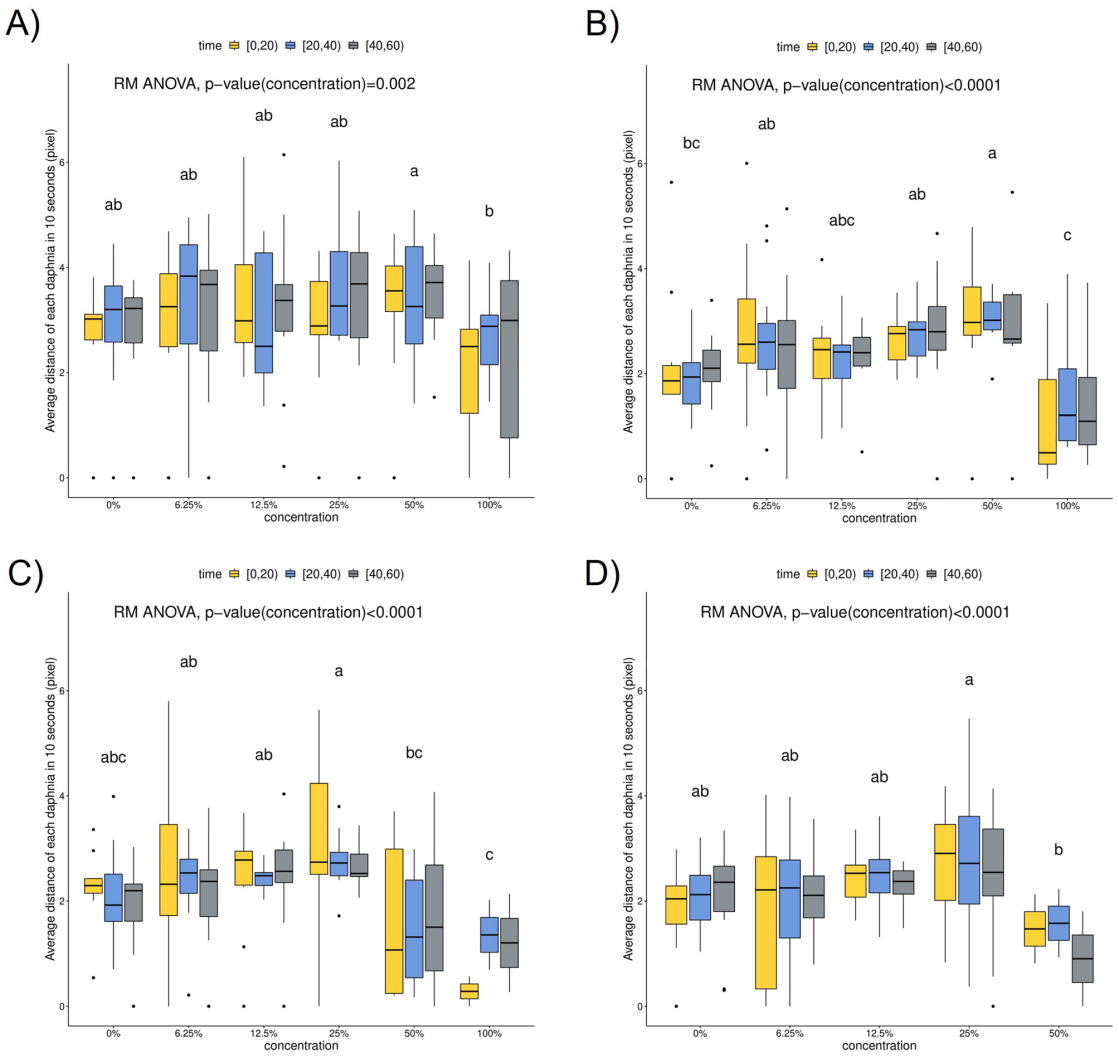
Box plot and RMANOVA results at 0, 12, 18, and 24 h. (**A**) P-values of interaction, time, and concentration effect at 0 h were 0.086, 0.904, and 0.002, respectively; (**B**) P-values of interaction, time, and concentration effect at 12 h were 0.435, 0.526, and <0.0001, respectively; (**C**) P-values of interaction, time, and concentration effect at 18 h were 0.349, 0.263, and <0.0001, respectively; (**D**) P-values of interaction, time, and concentration effect at 24 h were 0.694, 0.676, and <0.0001, respectively.

The RMANOVA results for Potassium dichromate at 0 h (Fig. 8A) showed that the interaction effect of concentration and time was not statistically significant ($p>0.05$). The time effect was not statistically significant ($p>0.05$). The locomotory responses of *Daphnia magna* revealed a significant difference between concentrations ($p=0.002 < 0.05$). The average distance for each *Daphnia magna* for 20 s differed with concentration, whereas the average distance remained unchanged for each time interval. The post-hoc test for concentration effect showed that the average distance for each *Daphnia magna* at 100% concentration significantly differed from the average distance at 50%, whereas it was similar to the average distance at the rest of the concentrations.

The RMANOVA results for Potassium dichromate at 12 h (Fig. 8B) showed that the interaction effect of concentration and time was not statistically significant ($p > 0.05$). Moreover, the time effect was not statistically significant ($p>0.05$). Once again, the locomotory responses of *Daphnia magna* revealed a significant difference between concentrations ($p<0.0001$). The average distance for 20 s for each *Daphnia magna* differed for each concentration, while the average distance remained unchanged for each time interval. The post-hoc test for the concentration effect showed that the average distance for each *Daphnia magna* at 100% concentration significantly differed from that at 50%, whereas the average distance at a concentration of 12.5% did not differ significantly from that at other concentrations.

The RMANOVA results for Potassium dichromate at 18 h (Fig. 8C) showed that the interaction effect of concentration and time was not statistically significant ($p > 0.05$). The time effect was not statistically significant ($p > 0.05$). Similar to the previous two iterations, the locomotory responses of *Daphnia magna* revealed a significant difference between concentrations ($p<0.0001$). The average distance for 20 s for each *Daphnia magna* differed for each concentration, whereas the average distance remained unchanged for each time interval. The post-hoc test for the concentration effect showed that the average distance for each *Daphnia magna* at 100% concentration significantly differed from that at 25%, whereas the average distance at 0% did not differ significantly from that at other concentrations.

Finally, since all *Daphnia magna* were dead at 100% concentration, the average distance for 20 s for each *Daphnia magna* at 100% concentration could not be measured after 24 h of exposure (Fig. 8D). The RMANOVA result for Potassium dichromate at 24 h showed that the interaction effect of concentration and time was not statistically significant ($p > 0.05$) and the time effect was not statistically significant ($p>0.05$). Similar to all the previous iterations, the locomotory responses of *Daphnia magna* revealed a significant difference between concentrations ($p<0.0001$). The average distance for 20 s for each *Daphnia magna* differed for each concentration, while the average distance did not vary for each time interval. The post-hoc test for concentration effect showed that the average distance for each *Daphnia magna* at 50% concentration significantly differed from that at 25%, whereas it was similar to that at the rest of the concentrations.

## Discussion

The aquatic environment is a crucial environmental medium in the ecosystem. It supports various lifeforms from bacteria to higher organisms and facilitates the circulation of essential elements such as nitrogen, phosphorus, and sulfur. Various chemicals can contaminate the aquatic environment and adversely affect the survival of aquatic organisms. To monitor aquatic contamination caused by chemicals, *Daphnia magna* can be used by evaluating the effects of the pollutants on the survival and mortality of the organisms. The aquatic toxicity test performed on *Daphnia magna* is typically used to assess water quality against the national regulatory ecotoxicity standards. For rapid aquatic toxicity testing, several studies conducted after 2007 employed high-throughput video tracking systems equipped with a video camera and tracking software^2,5,6^. However, most studies have focused only on building hardware with existing tracking software^28^, or on creating an efficient tracking software^18^. In this study, we present an approach that focuses on building an integrated system with multi-flow cell hardware and automatic tracking software.

One-cell systems that are widely adopted for use in existing biomonitoring systems can only observe 100% concentration. Thus, they can only serve as biological warning alarms. In the case of our monitoring system, the EC50 can be obtained by continuously exposing *Daphnia magna* to various concentrations by automatically diluting the water. In this manner, the degree of toxicity can be measured through the multi-flow cell. For real-time toxicity monitoring, our system can dilute the water in each chamber to the desired concentration in real time using the automatic dilution injection module. Consequently, our equipment can quickly measure the EC50 value and the behavioral patterns of *Daphnia magna* over time using the faster video tracking algorithm.

## Conclusion

The proposed device comprises a constant temperature module, a natural pseudo-light, a multi-flow cell, and an IDS imaging camera. The proposed tracking algorithm involves automatic subtraction, database construction, machine learning training, and *Daphnia magna* detection and tracking. In this study, the performance of the system was tested (Figs. 5, 6, 7, and Table 1), and the proposed tracking method with RF achieved the best IDF1, IDP, IDR, and IDs scores (Table 1) and best processing time (Fig. 6B). Twenty-four h *Daphnia magna* acute toxicity tests performed in the lab and using the device provided similar results (Fig. 7); therefore, the experiment using the device can potentially replace the lab experiment. Finally, to monitor the behavioral patterns of *Daphnia magna* over time, we performed the RMANOVA analysis. The results of the RMANOVA analysis showed that the average distance at 50% concentration changed dramatically. At 0 h, the distance at 50% differed significantly from that at 100%, but it was closer to that at 100% after 12 h. (Fig. 8). This result implies that *Daphnia magna* movements are affected by the concentration of Potassium dichromate. In conclusion, our work can provide a more efficient and faster automatic high-throughput system for ecotoxicity testing using *Daphnia magna*. The proposed method can efficiently analyze behavioral patterns of organisms over time for different degrees of toxicity using the multi-flow cell of our monitoring system. Therefore, the high-throughput video tracking system developed in this study can significantly contribute to monitoring the toxic effects of chemicals in aquatic environments around industrial plants. Since the automatic tracking system does not load the GPU hardware, deep learning detection methods were not efficient in our system. Thus, we used RF and SVM as the detection method for efficient and fast detection of *Daphnia magna*. In future work, we will develop a system with GPU hardware and hope to use deep learning methods such as Faster R-CNN^44^, YOLO^45–47^, SSD^48^, and DETR^49^ for better detection performance. In this context, we also propose FairMOT, which is an integrated model for object detection and tracking^50^.

## Data Availability

The datasets used and/or analyzed during the current study are available from the corresponding author upon reasonable request.

## References

[1] Liu Z, Malinowski CR, Sepúlveda MS (2021). Emerging trends in nanoparticle toxicity and the significance of using daphnia as a model organism. Chemosphere.

[2] Häder D-P, Erzinger GS (2017). Daphniatox-online monitoring of aquatic pollution and toxic substances. Chemosphere.

[3] Jeong T, Jeon J, Kim S (2014). Development and evaluation of new behavioral indexes for a biological early warning system using daphnia magna. Drink. Water Eng. Sci..

[4] Nikitin, O., Nasyrova, E., Kalinina, A., Sadykova, K. & Latypova, V. Effect of various temperature and light intensity regimes on daphnia magna swimming behaviour. In *Conference: 19th SGEM International Multidisciplinary Scientific GeoConference EXPO Proceedings*, Vol. 19 (2019).

[5] Lovern SB, Strickler JR, Klaper R (2007). Behavioral and physiological changes in daphnia magna when exposed to nanoparticle suspensions (titanium dioxide, nano-c60, and c60hxc70hx). Environ. Sci. Technol..

[6] Huang Y, Campana O, Wlodkowic D (2017). A millifluidic system for analysis of daphnia magna locomotory responses to water-born toxicants. Sci. Rep..

[7] Spink A, Tegelenbosch R, Buma M, Noldus L (2001). The ethovision video tracking system-a tool for behavioral phenotyping of transgenic mice. Physiol. Behav..

[8] Henry J, Rodriguez A, Wlodkowic D (2019). Impact of digital video analytics on accuracy of chemobehavioural phenotyping in aquatic toxicology. PeerJ.

[9] Wang X, Cheng E, Burnett IS, Huang Y, Wlodkowic D (2017). Automatic multiple zebrafish larvae tracking in unconstrained microscopic video conditions. Sci. Rep..

[10] Wang, X., Cheng, E., Burnett, I. S., Wilkinson, R. & Lech, M. Automatic tracking of multiple zebrafish larvae with resilience against segmentation errors. In *2018 IEEE 15th International Symposium on Biomedical Imaging (ISBI 2018)*, 1157–1160 (IEEE, 2018).

[11] Branson K, Robie AA, Bender J, Perona P, Dickinson MH (2009). High-throughput ethomics in large groups of drosophila. Nat. Methods.

[12] Chenouard N (2014). Objective comparison of particle tracking methods. Nat. Methods.

[13] Park J (2022). Acute adverse effects of metallic nanomaterials on cardiac and behavioral changes in *Daphnia magna*. Environments.

[14] Oppenheim, D. Object recognition for agricultural applications using deep convolutional neural networks. Ph.D. thesis, Master’s Thesis, Ben-Gurion University of the Negev, Beer-Sheva, Israel (2018).

[15] Song S, Li Y, Huang Q, Li G (2021). A new real-time detection and tracking method in videos for small target traffic signs. Appl. Sci..

[16] Huang, Y.-C., Liao, I.-N., Chen, C.-H., İk, T.-U. & Peng, W.-C. Tracknet: A deep learning network for tracking high-speed and tiny objects in sports applications. In *2019 16th IEEE International Conference on Advanced Video and Signal Based Surveillance (AVSS)*, 1–8 (IEEE, 2019).

[17] Yaseen ZM (2021). An insight into machine learning models era in simulating soil, water bodies and adsorption heavy metals: Review, challenges and solutions. Chemosphere.

[18] Bruijning M, Visser MD, Hallmann CA, Jongejans E (2018). trackdem: Automated particle tracking to obtain population counts and size distributions from videos in r. Methods Ecol. Evol..

[19] Bhagat SK, Tung TM, Yaseen ZM (2020). Development of artificial intelligence for modeling wastewater heavy metal removal: State of the art, application assessment and possible future research. J. Clean. Prod..

[20] Cho Y, Jonas-Closs RA, Yampolsky LY, Kirschner MW, Peshkin L (2022). Intelligent high-throughput intervention testing platform in *Daphnia*. Aging Cell.

[21] Dicle, C., Camps, O. I. & Sznaier, M. The way they move: Tracking multiple targets with similar appearance. In *Proceedings of the IEEE International Conference on Computer Vision*, 2304–2311 (2013).

[22] Rezatofighi, S. H. *et al.* Joint probabilistic data association revisited. In *Proceedings of the IEEE International Conference on Computer Vision*, 3047–3055 (2015).

[23] Kim, C., Li, F., Ciptadi, A. & Rehg, J. M. Multiple hypothesis tracking revisited. In *Proceedings of the IEEE International Conference on Computer Vision*, 4696–4704 (2015).

[24] Bewley, A., Ge, Z., Ott, L., Ramos, F. & Upcroft, B. Simple online and realtime tracking. In *2016 IEEE International Conference on Image Processing (ICIP)*, 3464–3468 (IEEE, 2016).

[25] Liu, K.-C., Shen, Y.-T. & Chen, L.-G. Simple online and realtime tracking with spherical panoramic camera. In *2018 IEEE International Conference on Consumer Electronics (ICCE)*, 1–6 (IEEE, 2018).

[26] Menshov, S., Wang, Y., Zhdanov, A., Varlamov, E. & Zhdanov, D. Simple online and realtime tracking people with new “soft-iou” metric. In *AOPC 2019: AI in Optics and Photonics*, Vol. 11342, 113420M (International Society for Optics and Photonics, 2019).

[27] Wojke, N., Bewley, A. & Paulus, D. Simple online and realtime tracking with a deep association metric. In *2017 IEEE International Conference on Image Processing (ICIP)*, 3645–3649 (IEEE, 2017).

[28] Simão FC (2019). Using a new high-throughput video-tracking platform to assess behavioural changes in daphnia magna exposed to neuro-active drugs. Sci. Total Environ..

[29] Korea national institute of environmental research: Korea official test method-water pollution, es 04704.1b (2017).

[30] Gonzales-Barron U, Butler F (2006). A comparison of seven thresholding techniques with the k-means clustering algorithm for measurement of bread-crumb features by digital image analysis. J. Food Eng..

[31] Hartigan JA (1975). Clustering Algorithms.

[32] Kittler J (1983). On the accuracy of the Sobel edge detector. Image Vis. Comput..

[33] Bosch, A., Zisserman, A. & Munoz, X. Image classification using random forests and ferns. In *2007 IEEE 11th International Conference on Computer Vision*, 1–8 (IEEE, 2007).

[34] Thai LH, Hai TS, Thuy NT (2012). Image classification using support vector machine and artificial neural network. Int. J. Inf. Technol. Comput. Sci..

[35] Safavian SR, Landgrebe D (1991). A survey of decision tree classifier methodology. IEEE Trans. Syst. Man Cybern..

[36] Zhou, D. et al. Iou loss for 2D/3D object detection. In *2019 International Conference on 3D Vision (3DV)*, 85–94 (IEEE, 2019).

[37] Wang L, Zhang Y, Feng J (2005). On the Euclidean distance of images. IEEE Trans. Pattern Anal. Mach. Intell..

[38] Shin Y (2019). The prediction of diatom abundance by comparison of various machine learning methods. Math. Probl. Eng..

[39] Leal-Taixé, L., Milan, A., Reid, I., Roth, S. & Schindler, K. Motchallenge 2015: Towards a benchmark for multi-target tracking. arXiv preprint arXiv:1504.01942 (2015).

[40] Milan, A., Leal-Taixé, L., Reid, I., Roth, S. & Schindler, K. Mot16: A benchmark for multi-object tracking. arXiv preprint arXiv:1603.00831 (2016).

[41] Li L, Sun F, Liu Q, Zhao X, Song K (2021). Development of regional water quality criteria of lead for protecting aquatic organism in Taihu Lake, China. Ecotoxicol. Environ. Saf..

[42] Hertzog C, Rovine M (1985). Repeated-measures analysis of variance in developmental research: Selected issues. Child Dev..

[43] de Mendiburu, F. & de Mendiburu, M. F. Package ‘agricolae’. R Package, Version 1–2 (2019).

[44] Ren S, He K, Girshick R, Sun J (2015). Faster r-cnn: Towards real-time object detection with region proposal networks. Adv. Neural. Inf. Process. Syst..

[45] Redmon, J., Divvala, S., Girshick, R. & Farhadi, A. You only look once: Unified, real-time object detection. In *Proceedings of the IEEE Conference on Computer Vision and Pattern Recognition*, 779–788 (2016).

[46] Redmon, J. & Farhadi, A. Yolo9000: Better, faster, stronger. In *Proceedings of the IEEE Conference on Computer Vision and Pattern Recognition*, 7263–7271 (2017).

[47] Redmon, J. & Farhadi, A. Yolov3: An incremental improvement. arXiv preprint arXiv:1804.02767 (2018).

[48] Liu, W. et al. Ssd: Single shot multibox detector. In *European Conference on Computer Vision*, 21–37 (Springer, 2016).

[49] Carion, N. et al. End-to-end object detection with transformers. In *European Conference on Computer Vision*, 213–229 (Springer, 2020).

[50] Zhang, Y., Wang, C., Wang, X., Zeng, W. & Liu, W. Fairmot: On the fairness of detection and re-identification in multiple object tracking. arXiv preprint arXiv:2004.01888 (2020).

